# Progressive multifocal leukoencephalopathy in the background of chlorambucil treated chronic lymphocytic leukemia: a case report from Nepal

**DOI:** 10.1097/MS9.0000000000001190

**Published:** 2023-08-14

**Authors:** Bishal Dhakal, Binit Upadhaya Regmi, Ram Chandra Subedi, Sushil Joshi, Bishnu Deep Pathak, Kanchan Bogati, Sunil Baniya, Peter Neupane, Raju Paudel

**Affiliations:** a Nepalese Army Institute of Health and Sciences, College of Medicine; b Cgrandy International Hospital; c Dodhara Primary Health Care Center; d Patan Academy of Health Sciences, Kathmandu; e Jibjibe Primary Health Care Center, Rasuwa, Nepal

**Keywords:** chlorambucil, chronic lymphocytic leukaemia, immunosuppression, prognosis, progressive multifocal leukoencephalopathy

## Abstract

**Introduction:**

Progressive multifocal leukoencephalopathy is a rare manifestation in itself. Although many immunosuppressive states are associated with the disease, its occurrence in the setting of chronic lymphocytic leukaemia treated with chemotherapy is seldom reported to date.

**Case presentation:**

A 67-year-old woman with known chronic lymphocytic leukaemia who was previously receiving chlorambucil treatment was identified as having progressive multifocal leukoencephalopathy; her prognosis is currently good.

**Clinical discussion:**

Although a rare disease in an immunocompromised setting, progressive multifocal leukoencephalopathy often leads to a grave outcome. However, the authors describe a case with a good prognosis to date.

**Conclusion:**

Progressive multifocal leukoencephalopathy should be in differentials in immunocompromised patients with dementia. Given that the later prognosis of the disease is unpredictable, an earlier diagnosis would be better for immunological reconstitution.

## Introduction

Highlights

- Progressive multifocal leukoencephalopathy is a rare manifestation in an immunocompromised setting.

- Although it often leads to a grave prognosis, we describe a case with a good prognosis to date.

- Progressive multifocal leukoencephalopathy should be sought in patients with dementia even in developing countries.

Progressive multifocal leukoencephalopathy (PML), a rare demyelinating infection of the central nervous system (CNS) is caused by the JC polyomavirus^1–3^. It is an opportunistic viral infection that was first described in a patient with chronic lymphocytic leukaemia (CLL) by Åström *et al*.^4^ in 1958. The necessary condition for PML is an immunosuppressive state with suppressed cell-mediated immunity like HIV or lymphoproliferative malignancies, or treatment with immunosuppressive or immunomodulatory therapies^2^. It has been shown to be associated with various haematological malignancies like chronic lymphocytic leukaemia, acute myeloid leukaemia, mantle cell lymphoma, acute lymphoblastic leukaemia, Hodgkin’s lymphoma, and those receiving chemotherapies^5^. The clinical features of PML vary depending on the brain lesion in corresponding areas. The presenting symptoms may include hemiparesis, sensory deficit, visual deficits, cognitive dysfunction, aphasia, or coordination and gait difficulties^2,3^.

Here, we present a case of a 67-year-old female with progressive multifocal leukoencephalopathy who presented with abnormal behaviour and forgetfulness in the background of completion of chemotherapy with Chlorambucil for CLL 2 years back. This case report is reported based on the SCARE guidelines^6^.

## Case report

**Figure 1:**
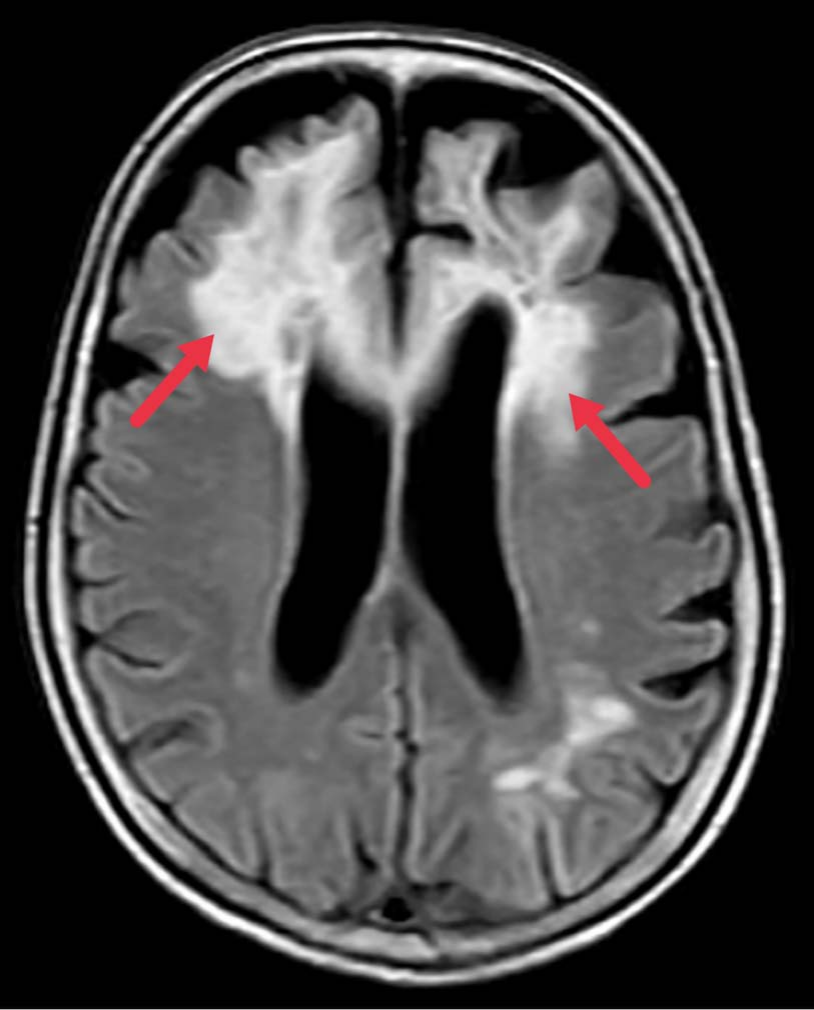
Magnetic resonance imaging head showing periventricular and subcortical white matter changes (red arrows).

A 67-year-old female, a known case of CLL and hypertension, presented to our medical facility with abnormal behaviours, forgetfulness, screaming at others, and loss of appetite for 2 months. She used to forget the last meals taken, the names of the family members, and even forget her room. The abnormal behaviours included screaming at others and shouting for no cause. She had no fever, headache, vomiting, abnormal body movements, diplopia, and focal neurological deficits. She was given chemotherapy with a standard dose of six cycles of Chlorambucil (0.5 mg/kg) 2 years back for CLL while she was abroad and was on haematological remission since then. On examination, she appeared cachectic and pallor was present. On neurological evaluation, immediate memory and serial subtraction test were impaired. Her mini-mental status examination score was 20 out of 30. Other general and systemic findings were within normal limits. The routine investigations showed haemoglobin 9.4 g/dl (13–17 g/dl), total count 14870/mm^3^ (4000–1100/mm^3^) with 77% lymphocytes (20–40%) and 16% neutrophils (40–60%). Total protein and albumin were 4.9 g/dl (5.5–9 g/dl) and 3.7 g/dl (3.5–5.5 g/dl), respectively. Serology was negative for HIV 1, 2, HBsAg, and Anti HCV. Magnetic resonance imaging (MRI) brain was ordered as shown in Figure 1.

It showed bilateral asymmetrical signal changes involving the periventricular and subcortical white matter of bilateral frontal lobes with involvement of the genu and anterior body of the corpus callosum, left parieto-temporal lobes, right parietal lobe, and right cerebellar hemisphere with mild age-related atrophy. Based on the above history, clinical findings, and laboratory investigations, she was admitted to our hospital for further evaluation and management. She was managed as a case of toxic leukoencephalopathy with CLL and hypertension. The cerebrospinal fluid (CSF) tapping was done and sent for relevant investigations. CSF analysis showed a total Count- less than 5 cells with all lymphocytes, glucose 52 mg/dl (40–80 mg/dl), protein 10 mg/dl (20–40 mg/dl), adenine deaminase level 2.83 U/L (<10 U/L). CSF neurotropic viral panel came out positive. CSF for JC was sent before discharge to be followed up during the OPD visits.

During discharge, she still had problems with memory, and was advised to increase family participation. As there is no definite cure for PML, the patient was discharged on multivitamin supplementation and anticholinergic (Donepezil 5 mg once daily). On the follow-up visit, polymerase chain reaction (PCR) for the JC virus in CSF showed 400 000 copies /ml, and a diagnosis of PML with JC virus was made. Although there are no any established criteria for the diagnosis of PML, we came to the diagnosis based on the neurological deficits in an immunocompromised state with the help of MRI findings and PCR of the CSF from the patient. During the follow-up out-patient department visit after 1 month, there was no further deterioration in the general and neurological condition of the patient.

## Discussion

Progressive multifocal leukoencephalopathy is a rare disease occurring almost exclusively in immunosuppressed patients^2^. There are few population-based studies on PML epidemiology, the most recent is from Southern Finland in 2004–2016, which reported a PML incidence of 0.12/100 000 person-years^7^. A Swedish study found that PML was most commonly associated with malignancy (42%), with haematological malignancies being the most common, followed by HIV/AIDS (31%), and autoimmune disease (21%). 57% of PML (HIV/AIDS excluded) have a history of immunosuppressive or chemotherapeutic treatment (current or prior treatment within the last 12 months), making it a significantly associated risk factor^8^. Immunomodulatory agents such as rituximab and natalizumab, as well as chemotherapeutic agents, are frequently implicated^8^. From the literature, it is clear that profound cellular immunosuppression is the major risk factor that allows virus reactivation and the development of the disease, which was primarily acquired during early childhood as an asymptomatic infection^2^. In order to establish the central nervous system infection, the JC polyomavirus must be reactivated after the primary infection (through the respiratory route). It mostly remains in the kidney as a latent phase. In the immunocompromised setting, the reactivated virus rearranges into the neurotropic strain and infects the oligodendrocytes. And with deranged immunosurveillance, the virus undergoes uncontrolled replication and manifests as PML^9^.

Classic PML symptoms include altered mental status, motor deficits (hemiparesis or mono paresis), limb ataxia, and visual symptoms (hemianopsia or diplopia)^10^; however, in the early stages, it may be asymptomatic^11^. Initial symptoms depend upon the location of the lesion in the CNS white matter^12^. As per a recent nationwide study in Sweden on PML incidence, there is evidence that 50% of PML patients have cognitive deficits^8^. In Nepal, dementia is the most common diagnosis (12.5%) among organic mental disorders in elderly patients (65 years of age and older) presenting to the psychiatric outpatient department of a tertiary care centre, as per a retrospective review^13^. Because our patient was also found to have cognitive deficits, she was evaluated for PML as well. A clinician should suspect PML in patients who present with subacute neurologic deficits in the setting of immunosuppression or immunomodulatory therapy if brain MRI reveals focal or multifocal white matter lesions, generally, without mass effect, that do not conform to vascular territories, and they should be evaluated with CSF analysis using PCR for the presence of JCV DNA, which establishes the diagnosis. Although brain biopsy is the gold standard, it is considered only in circumstances where other conditions like CNS lymphoma are part of the differential diagnosis^10^.

PML is itself a rare disease and is also one of the rare complications of immunosuppressive chemotherapy^14^. Our case is most likely the first case of PML related to chlorambucil used for the treatment of CLL to be reported from Nepal. Our patient presented with 2 months of symptoms after 2 years of chemotherapy-induced CLL remission and was thus diagnosed, which is consistent with the usual median period of 2.1 months from symptom onset to diagnosis^5^. Chlorambucil is an alkylating agent used to treat CLL, Hodgkin lymphoma, and autoimmune diseases, which is observed to be associated with PML as reported by a few other studies^8,15,16^.

The outcome of PML likely depends on how previous haematological malignancies were treated. PML cases arising in the background of hematopoietic stem cell transplantation have a longer time to diagnose, better survival rates, and lower mortality rates as compared to cases arising in the background of chemotherapy or immunotherapy. This study shows a median survival of 2 months in PML cases related to chemo or immunotherapy^14^. Currently, there is no specific prophylaxis and no effective treatment for PML; however, the best approach is prompt immune reconstitution without causing immune reconstitution inflammatory syndrome^2^. And this can be done by supportive measures and by stopping the immunomodulatory therapy^5,9^.

## Conclusion

Presentation of dementia in neurology OPD is quite common. Regarding this case report, we would like to highlight the need for JC virus PCR in CSF in patients with immunosuppressants or those receiving immune-modulatory therapy. In our part of the world, testing JCV is not done in many cases of dementia due to a lack of expertise or financial issues. Early detection and care of PML are crucial because the prognosis depends on the associated comorbidities and timing of the diagnosis. The differentials should be broad in such cases presenting with the typical MRI findings in the background of the use of chemotherapy or immunosuppressive agents.

## Author agreement statement

We the undersigned declare that this manuscript is original, has not been published before and is not currently being considered for publication elsewhere.

We confirm that the manuscript has been read and approved by all named authors and that there are no other persons who satisfied the criteria for authorship but are not listed. We further confirm that the order of authors listed in the manuscript has been approved by all of us.

We understand that the Corresponding Author is the sole contact for the Editorial process. He/she is responsible for communicating with the other authors about progress, submissions of revisions and final approval of proofs.

## Ethical approval

This is a case report, therefore, it did not require ethical approval from ethics committee.

## Consent

Written informed consent was obtained from the patient for publication of this case report and accompanying images. A copy of the written consent is available for review by the editor-in-chief of this journal on request.

## Sources of funding

The study did not receive any grant from funding agencies in the public, commercial or not-for-profit sectors.

## Author contribution

B.D.: conceptualization, writing—original draft. R.C.S., B.U.R. and B.D.P.: conceptualization, writing—review and editing. S.J., K.B., S.B. and P.N.: resources, validation, investigation. R.P.: conceptualization, supervision. All the authors read and approved the final manuscript.

## Conflicts of interest disclosure

The authors declare that they have no conflicts of interest.

## Research registration unique identifying number (UIN)

Not applicable.

## Guarantor

Bishal Dhakal.

## Data availability statement

Not applicable.

## Provenance and peer review

Not commissioned, externally peer-reviewed.
